# Ileal Diverticulitis

**DOI:** 10.5334/jbsr.3078

**Published:** 2023-02-20

**Authors:** Kelly Di Dier, Stephen Van Meerbeeck

**Affiliations:** 1 UZ Ghent, BE; 2 Radiologie Dokters Van Meerbeeck bv, BE

**Keywords:** ileum, diverticulitis, terminal ileal diverticulitis, ultrasound, computed tomography

## Abstract

**Teaching Point:** Terminal ileal diverticulitis is an uncommon cause of right lower abdominal pain.

## Case History

**Figure 1:**
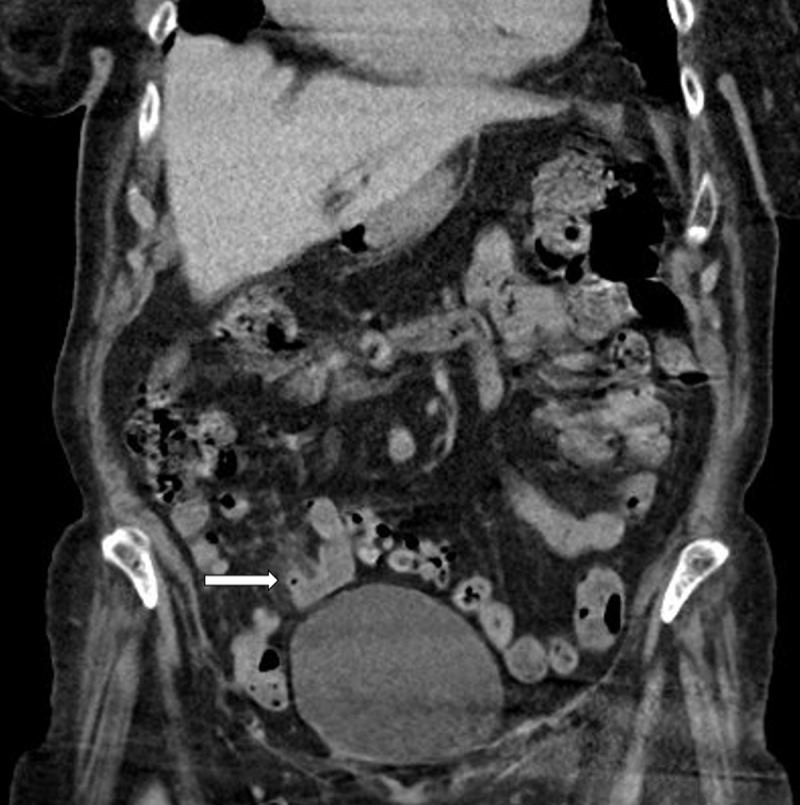


**Figure 2:**
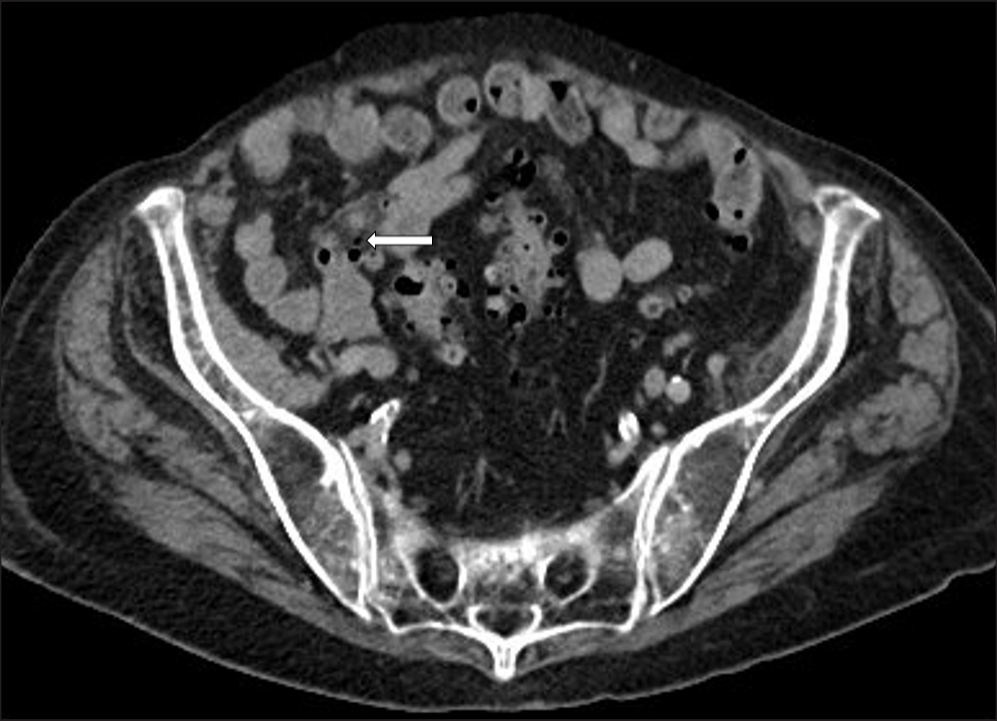


**Figure 3:**
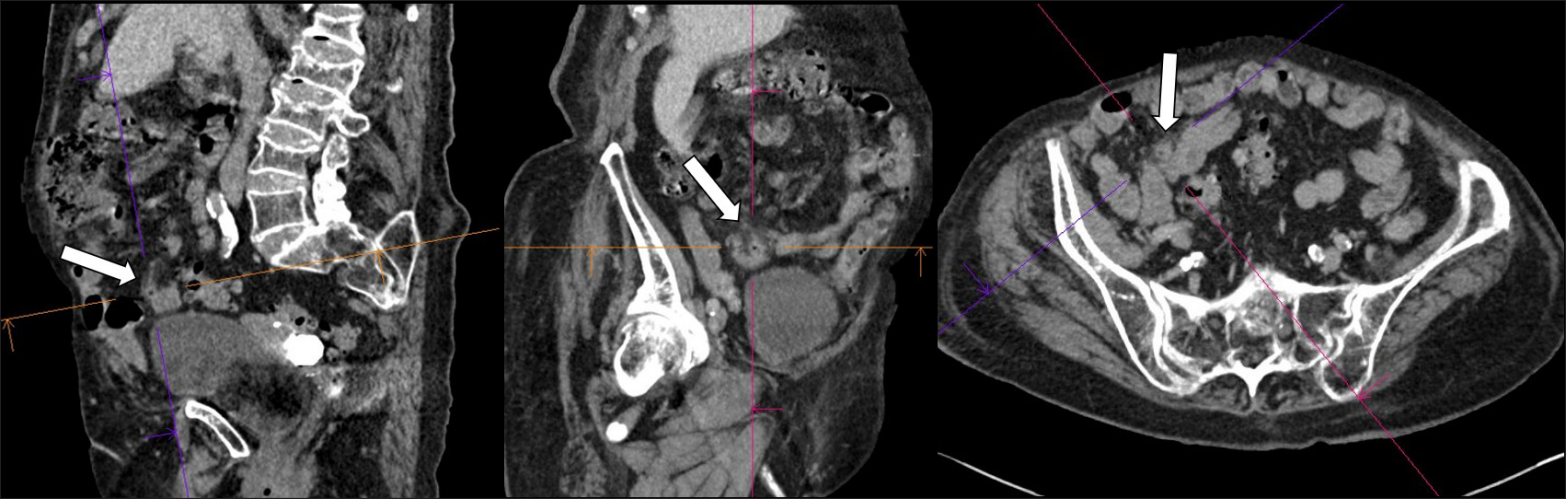


A 92-year-old female presented at the emergency department with acute abdominal pain in the right lower quadrant. Blood analysis revealed elevated CRP and leukocytosis. The emergency doctor suspected acute appendicitis. Abdominal ultrasound was performed with inconclusive results. Subsequent contrast-enhanced computed tomography (CT) of the abdomen was executed. An inflamed, wall-thickened diverticulum was present in the terminal ileum, corresponding with terminal ileal diverticulitis (Figure 1, arrow). A small adjacent air bubble was noted, compatible with a contained perforation (Figure 2, arrow). There was concomitant densification and stranding of the mesenteric fat, corresponding to localised mesenteritis (Figure 3, arrow). There were no signs of non-contained perforation or circumjacent abscess.

## Comments

Diverticula are a well-known entity in the colon. When present in the small bowel, the duodenum and less frequently the jejunum are affected. Ileal diverticula, more specifically, terminal ileal diverticula are rare. Small bowel diverticula are observed twice as often in men than in females, with a peak incidence between the sixth and seventh decade [1].

Small bowel diverticulosis is often asymptomatic. When inflamed, these terminal ileal diverticula cause acute abdominal pain in the right lower quadrant [1]. Diverticulitis can be associated with a contained perforation and localised peritonitis of the small bowel mesentery. Other pathological entities in terminal ileal diverticulosis are bleeding, frank perforation, or obstruction, but these are even more rare [1].

As in this case, terminal ileal diverticulitis is a mimicker of acute appendicitis. Other causes of right lower quadrant pain that are more frequently observed than terminal ileal diverticulitis are right-sided colon diverticulitis and inflammation due to Crohn’s disease. Meckel diverticulitis and appendagitis epiploica are less frequently observed differential diagnoses. Differentiation between these diagnoses is based on imaging studies. Ultrasound is usually the first imaging method, due to its accessibility and low cost. Sac-like appendages on the ileum with wall thickening can be demonstrated. Hyperechoic surrounding fat tissue indicates inflammation. Nonetheless, CT is the imaging modality of choice as it is more capable of examining the pathological entities. Diverticula of the terminal ileum are well demonstrated. When inflamed, wall thickening is effortlessly observed, as shown in this case. Contained perforation can be seen as an adjacent air bubble, but it is not always found. The associated fat stranding of the surrounding tissue is seen easily [1]. CT is even more useful in examining possible complications, such as non-contained perforation and abscesses.

Therapy is usually conservative [1]. Antibiotics and analgaesics are mostly sufficient. Surgery is performed when complications occur [1].

Terminal ileal diverticulitis mimics appendicitis as a rare cause of right lower quadrant pain in the abdomen.
